# Frontal stimulation reshapes SCAN connectivity and improves motor function in Parkinson’s disease

**DOI:** 10.64898/2026.08.31.748359

**Published:** 2026-09-04

**Authors:** Rupsha Panda, James A. Brissenden, Teresa Scerbak, Erin Proctor, Yiran Li, Michael Vesia, Roger L. Albin, Taraz G. Lee

## Abstract

Parkinson’s disease is a neurodegenerative disorder with characteristic motor deficits of bradykinesia and impaired coordination, and non-motor deficits affecting cognitive abilities such as attention and executive functions. Recent evidence suggests that some canonical motor deficits in Parkinson’s disease (e.g., freezing of gait) are associated with abnormalities in frontal cortical processing typically thought to underlie executive functions such as cognitive control. The evidence from human research linking frontal cognitive disruption and motor deficits in people with Parkinson’s disease is correlational. Individuals with cognitive impairments often also display gait and balance deficits, but causal evidence remains lacking. We combined non-invasive brain stimulation with functional MRI (fMRI) to ascertain whether dorsolateral prefrontal cortex (DLPFC) exerts a causal influence modulating motor function and to test the hypothesis that an excitatory stimulation protocol applied to a cognitive control network can improve motor performance in Parkinson disease.

Participants with Parkinson’s disease performed a precision force-tracking task shown to track with gait impairment while undergoing functional neuroimaging scans. On half of trials, participants were required to simultaneously perform a cognitively demanding 2-back working memory task to tax attentional systems. Following the initial baseline scan, participants returned for three separate counterbalanced sessions that combined transcranial magnetic stimulation (TMS) in the form of theta burst stimulation (TBS) with fMRI. Just prior to task performance, participants received either excitatory (intermittent TBS) or inhibitory (continuous TBS) stimulation protocols over right DLPFC. In the third session, participants received stimulation to a control site outside the cognitive and motor areas implicated in our task. Our within-subjects experimental design allowed us to examine the effects of stimulation on motor behavior, the influence of cognitive load on motor behavior, and the neural systems supporting cognitive control and motor execution.

Consistent with our hypothesis, excitatory stimulation of DLPFC improved motor performance in a precision force-tracking task as indexed by reduced tracking error and smoother movement execution. These effects emerged preferentially under dual-task conditions when cognitive demands were high. Our results show that this improved motor performance coincides with reductions in functional connectivity not only between the stimulation site and primary motor cortex, but also within the recently described somato-cognitive action network (SCAN) thought to support action planning and whole-body movement coordination.

We conclude that frontal cognitive control systems play a causal role in modulating movement in Parkinson’s disease and suggest that targeting these systems with non-invasive interventions may enhance motor function.

## Full Text Availability

The license terms selected by the author(s) for this preprint version do not permit archiving in PMC. The full text is available from the preprint server.

